# The cytogenetics of Bloom’s syndrome

**DOI:** 10.4103/1817-1745.76124

**Published:** 2010

**Authors:** Ashish Singh, S. Ambujam, A. N. Uma

**Affiliations:** Departments of Dermatology, Venereology, Leprology, Mahatma Gandhi Medical College and Research Institute, Pillaiyarkuppam, Pondicherry - 607 402, India; 1 Anatomy Mahatma Gandhi Medical College and Research Institute, Pillaiyarkuppam, Pondicherry - 607 402, India

Sir,

Bloom’s syndrome is an autosomal recessive disorder characterized by distinctive faces, stunted growth, telangiectatic facial erythema, abnormal immune response and predisposition to various malignancies. Cytogenetically, it is characterized by increased frequency of spontaneous sister chromatid exchange.

**Figure 1:**
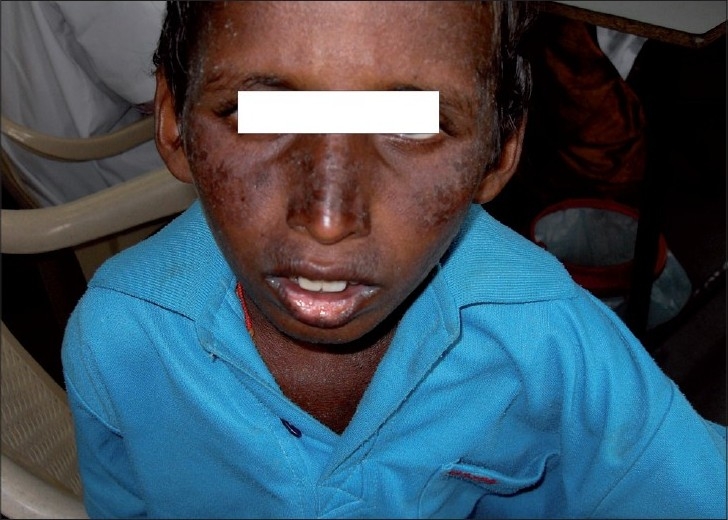
Patient of Bloom’s syndrome showing scaly plaques over malar area

A 10-year-old boy born of third degree consanguineous parents was referred by pediatricians with complaints of rashes over face which started 6 months after birth. History suggestive of photosensitivity was present. Examination revealed erythematous scaly plaques with telangiectasia over butterfly area of face, neck, ears, and lower lip (mainly over the sun-exposed areas) [Figure 1]. The child also had prominent nose, narrow and slender faces. His physical growth was stunted. On the basis of the above mentioned features, we diagnosed Bloom’s syndrome in this patient.

**Figure 2:**
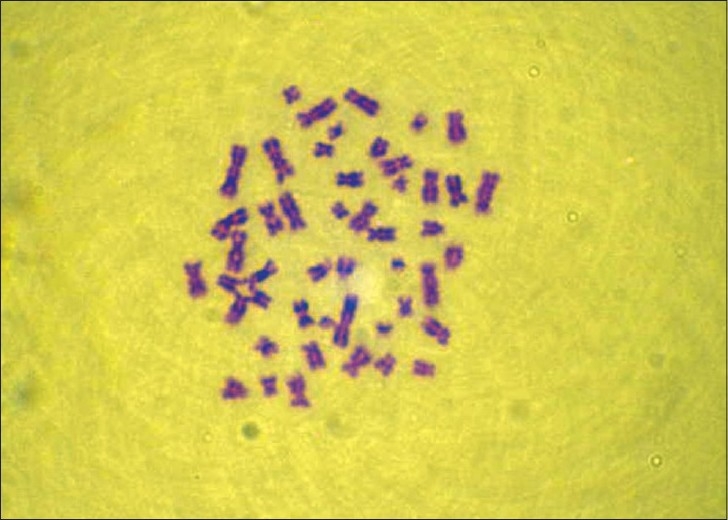
Quadriradial figure of chromosome

**Figure 3:**
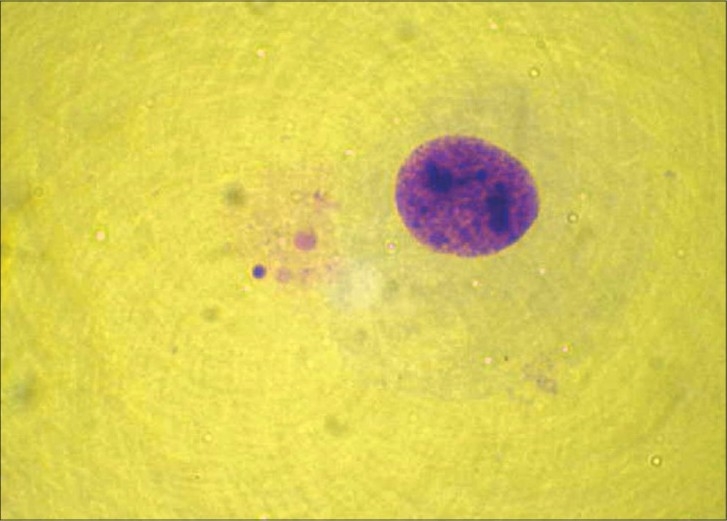
Micronucleus detected

**Figure 4:**
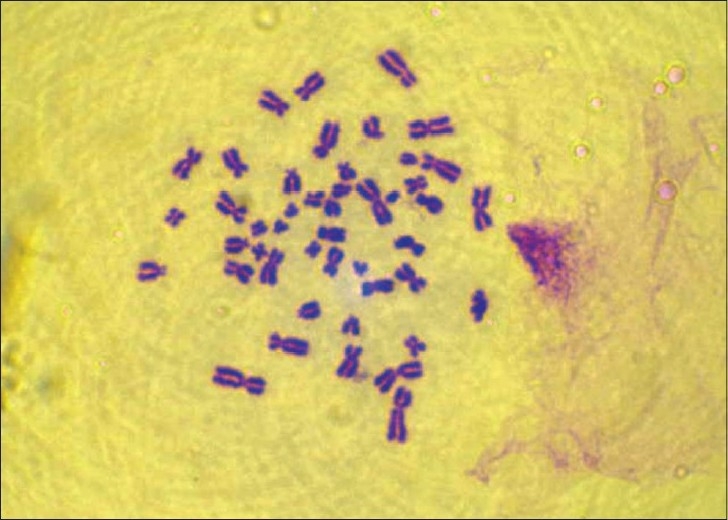
Chromosomal fragments

Cytogenetic study was done for the patient. Leukocyte culture of the individual showed a normal karyotype 46 XY but with 20% aberrant metaphases with chromosomes showing breaks, fragments, and micronucleus [Figures 2,3]. Fifty metaphases were analyzed. The frequency of satellite association (SA) was 32% [Figure 4]. Similar cytogenetic study was also done on four controls and results revealed average of only 11% SA.

**Figure 5:**
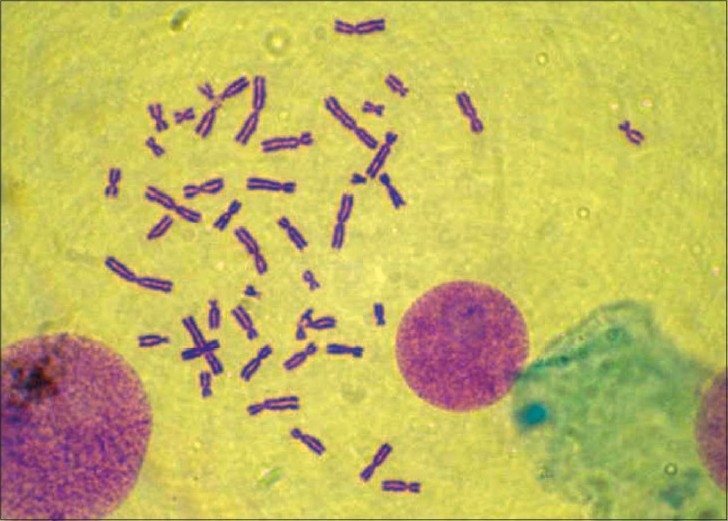
Acrocentric association of chromosomes

*In vitro* leukocyte culture of the same child when exposed to ultraviolet (UV) radiation showed 80% aberrant metaphase with chromosomes showing quadriradial figure, multiple breaks, gaps, fragments, and increased incidence of micronucleus [Figure 5]. The frequency of SA also increased to 84%, confirming that when there is an increased evidence of unstable chromosome on exposure to UV radiation, there is also an increased incidence of SA, an indicator of defect in the DNA repair mechanism.

In acrocentric association, satellite chromosomes, that is, chromosomes nos. 13, 14, 15, 21, and 22, come closer at the tip of short arm due to some sticky substance formed during meiosis. Acrocentric association has been detected earlier in some syndromes including Down’s syndrome.[1] Acrocentric association results in increased incidence of chromosomal rearrangements and later on, can cause chromosomal diseases.[2]

Chromosomal breakage and rearrangement occur spontaneously in three disorders namely Bloom’s syndrome, Fanconi’s anemia, and Ataxia Telangiectasia.[3] Bloom’s syndrome is characterized by the presence of quadriradial chromosomes.[4] These are chromosome with four arms, formed by recombination between two chromosomes. It is found only in 0.5 to 14% of cases. The diagnosis is confirmed by the demonstration of spontaneously enhanced formation of sister chromatid exchange.[4]

Micronucleus frequency in peripheral blood lymphocytes is extensively used in cytogenetics to evaluate the presence and extent of chromosomal damage.[5] It is regarded as the most sensitive and convenient method to detect chromosomal damage.[4] It is the result of chromosomal breakage due to malrepaired or unrepaired DNA lesion, or chromosomal malsegregation due to mitotic malfunction.[5] It originates from chromosome fragment or whole chromosome, not included in the main daughter nuclei during nuclear division.[5] Association between micronucleus induction and cancer development or cancer-prone congenital disease like Bloom’s syndrome has been supported by a number of observations.[5]

Authors feel that cytogenetic studies in genodermatoses like Bloom’s syndrome would help not only to prevent the severity of the disease with passage of time, but also to detect skin malignancies that are liable to occur.
